# Selecting an Invertebrate Model Host for the Study of Fungal Pathogenesis

**DOI:** 10.1371/journal.ppat.1002451

**Published:** 2012-02-02

**Authors:** Athanasios Desalermos, Beth Burgwyn Fuchs, Eleftherios Mylonakis

**Affiliations:** Division of Infectious Diseases, Massachusetts General Hospital, Harvard Medical School, Boston, Massachusetts, United States of America; Duke University Medical Center, United States of America

## Invertebrate Hosts Are a Valuable Tool in Studying Fungal Pathogens

The use of invertebrate hosts as infection models can greatly facilitate the study of pathogenesis (Table 1). Among invertebrate model hosts, the available options to select from include amoebae (*Acanthamoeba castellanii* and *Dictyostellium discoideum*) [1], [2], the nematode *Caenorhabditis elegans* [3], and insects (including *Drosophila melanogaster*, *Galleria mellonella*, and *Bombyx mori*) [4]–[8].

**Table 1:** Summary of findings generated by using the invertebrate infection models.

Model Host	*A. castellanii*	*C. elegans*	*D. discoideum*	*D. melanogaster*	*G. mellonella*
Types of fungi studied	*Blastomyces dermatitidis*, *Cryptococcus neoformans* [1], *Histoplasma capsulatum*, *Sporothrix schenckii*	*Saccharomyces cerevisiae*, *Candida albicans* [17], *Cryptococcus neoformans* [3], *Drechmeria coniospora*	*Cryptococcus neoformans* [2]	*Candida albicans* [4], *Cryptococcus neoformans* [18], *Aspergillus fumigatus*, *Beauveria bassiana*	*Candida albicans* [5], *Cryptococcus neoformans*, *Fusarium oxysporum*, *Aspergillus flavus*, *Aspergillus fumigatus*
Representative virulence factors studied on the model	*CAP67* (capsule related, *C. neoformans*) [1], melanin genes, (melanin related, *C. neoformans*) [1], *PLB* (phospholipase related, *C. neoformans*) [1]	*CAP59* (capsule related, *C. neoformans*) [3], *GPA1* (G protein alpha subunit related, *C. neoformans*) [3], *PKA1* (cAMP- dependent protein kinase subunit, *C. neoformans*) [3], *RAS1* (high temperature growth related, *C. neoformans*) [3], *LAC1* (related to melanin production, *C. neoformans*) [3], *ADE2* (phosphoribosylaminoimidazole related, *C. neoformans*) [3], *KIN1* (protein kinase related, *C. neoformans*), *ROM2* (Rho1 guanyl nucleotide exchange factor related, *C. neoformans*) [19], *RIM101* (hyphal formation related, *C. albicans*) [17], *NRG1* (hyphal formation related, *C. albicans*) [17], *CAS5* (hyphal formation related, zinc finger protein related, *C. albicans*) [17], *ADA2/CAS3* (hyphal formation related, *C. albicans*) [17]	*CAP67* (capsule related, *C. neoformans*) [2]	*CDC35* (adenylyl cyclase related, *C. albicans*) [4], *CLA4* (activated kinase related, *C. albicans*) [4], *SAP4-6* (aspartyl protease related, *C. albicans*) [4], *PKA1* (cAMP-dependent protein kinase subunit, *C. neoformans*) [18]	*CDC35* (adenylyl cyclase related, *C. albicans*) [5], *CLA4* (activated kinase related, *C. albicans*) [5], *CAP59* (capsule related, *C. neoformans*), *BCR1* (filamentation related, *C. albicans*) [20], *FLO8* (filamentation related, *C. albicans*) [20], *KEM1* (filamentation related, *C. albicans*) [20], *SUV3* (filamentation related, *C. albicans*) [20], *TEC1* (filamentation related, *C. albicans*) [20]

A critical step in addressing a question or hypothesis regarding host–pathogen interactions is to determine which infection model(s) best fit into the experimental criteria. For example, *Cryptococcus neoformans* and *Candida albicans* can infect amoebae, *C. elegans*, and several insect hosts. However, not all hosts are amenable to infection by every fungal pathogen, conditions for infections need to be optimized, and in some cases the host is not favorable for the study of the particular pathogenesis trait. For example, *Pneumocystis murina* cannot infect *D. melanogaster* or *G. mellonella*.

**Figure 1:**
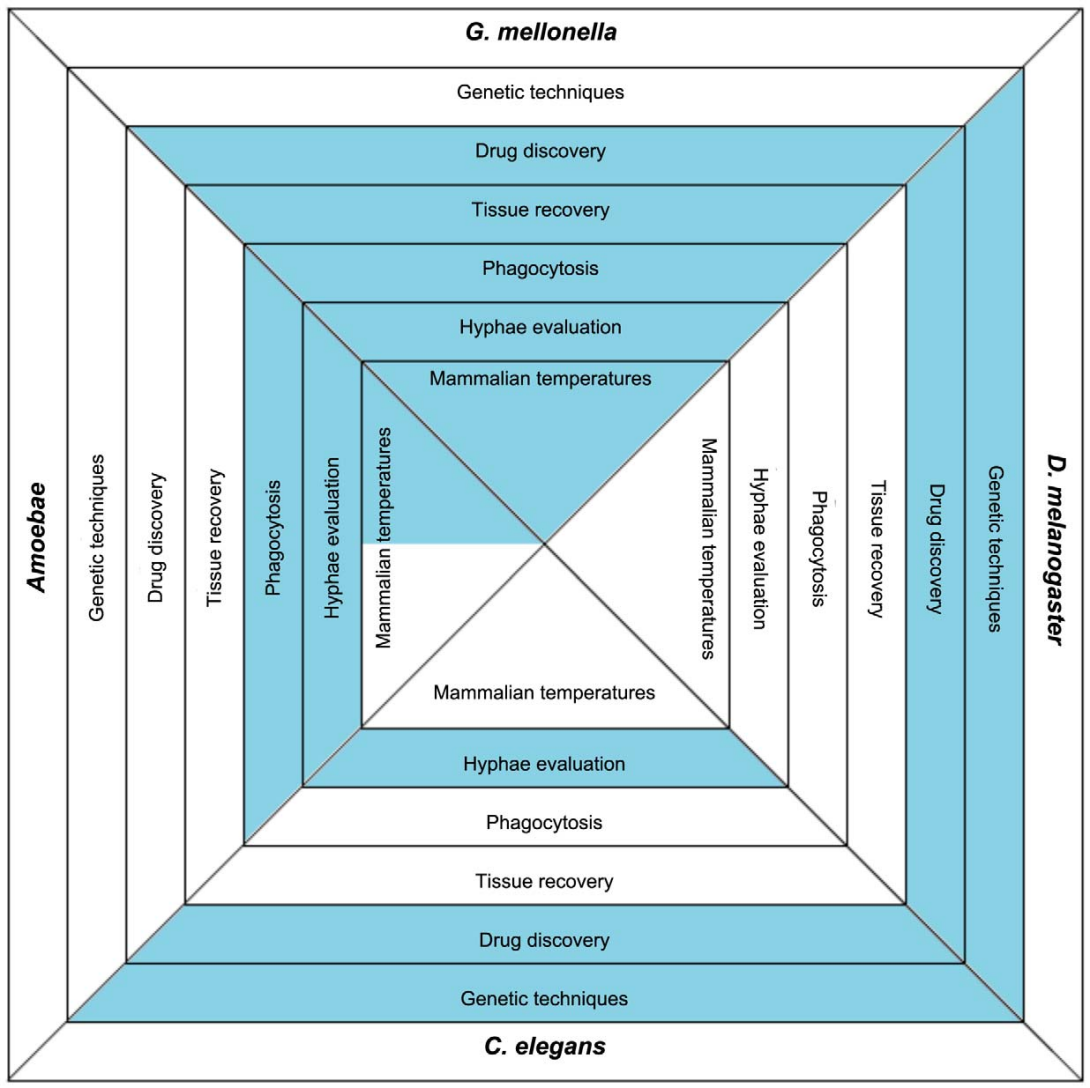
The basic characteristics of the more frequently used model hosts. The blue color indicates that this feature is found in the specific model host. Utilizing the features of the chart can aid in determining which host(s) are most amenable to a particular study. Host genetic tools aiding in understanding host–pathogen interaction include sequenced genomes, available mutant strains, or RNAi. Once infected, some hosts can be used to identify compounds with antifungal activity. Also, while infected, some hosts are large enough that individual portions or tissues from the hosts can be removed and further analyzed either for host responses or to evaluate tissue invasion by the pathogen. As part of the host response, some hosts have phagocytic cells that engulf the foreign fungi and can be studied to elucidate information about host–pathogen interactions. When some fungi are engulfed by phagocytes, or establish an infection within the hosts, they produce hyphae. Because of the transparency or ability to recover tissue from some of the hosts, fungal hyphae formation can be further evaluated. For all of the infecting pathogens, temperature conditions are a consideration. The various hosts have conditions that are ideal for meeting their own survival needs, and the fungi will react differently in terms of gene expression and growth rate based on the temperatures in which the hosts are maintained. Temperature features marked in grey on the chart indicate hosts that can survive at temperature ranges as high as 37°C. Other invertebrate model hosts including *Bombyx mori*, *Culex quinquefasciatus*, *Blattella germanica*, and even a plant model of *Arabidopsis thaliana* have been developed. They are not as widely used and not mentioned here in detail because of space limitations.

Available model hosts offer different advantages and disadvantages, and before choosing the right model host some basic questions should be posed: 1) are you interested in the host immune response to the infecting pathogen and what host-related tools, such as RNAi, sequenced genome, or mutants, are available and could be advantageous to such studies, 2) will the host be used for drug discovery, 3) will host tissue need to be removed and evaluated, 4) will host phagocytosis of the pathogen be studied, 5) is fungal hyphal formation of interest, and 6) what temperature conditions are best suited to address the research questions of interest or for studying a particular fungal gene (Figure 1).

## Are Virulence Traits Equally Important in All Systems?

The pathogenicity of fungi in mammals has many similarities with the pathogenicity in non-vertebrate hosts. Throughout their evolution, fungi have been in continuous contact and interaction with other soil-dwelling organisms, and it has been suggested that many virulent factors have evolved in order to protect fungi from environmental predators. However, all virulence traits are not equally important for the pathogenesis in different systems. For example, an intact capsule is critical for *C. neoformans* pathogenesis in *G. mellonella* and amoebae, but this is not the case in the killing of *C. elegans* [1], [3]. Therefore, the right choice of a model host is crucial for successful research.

## How to Study Temperature-Sensitive Virulence Traits?

Some, but not all, invertebrate hosts allow the study of pathogenesis at mammalian temperatures. For example, *D. melanogaster* and *C. elegans* are temperature restricted and cannot survive at high temperature testing conditions; *C. elegans* is better used at a temperature range from 15°C to 25°C, and *D. melanogaster* has an optimal temperature range from 18°C to 30°C. Although some model hosts have temperature ceilings that are lower than mammalian conditions, there are other more thermotolerant model hosts, such as some insects, including *G. mellonella* (which has a temperature range of 25°C to 37°C), some amoebae, or worms such as *Panagrellus redivivus* [9].

The higher thermotolerance presents conditions under which certain genes expressed at mammalian temperatures can be studied. However, mammalian temperatures are not always ideal for the study of a temperature-related trait. For example, multiple hosts were necessary to study *ECA1*, a sarcoplasmic/endoplasmic reticulum Ca^2+^-ATPase type calcium pump [10]. An *eca1 C. neoformans* mutant exhibited reduced growth at 37°C, so association with virulence was difficult to ascertain with mammalian models or by using *G. mellonella* or amoeba at 37°C conditions. A role in virulence was found using *G. mellonella* at 30°C and *C. elegans* at 25°C. This approach is interesting because using an array of model hosts has the advantage that we can study fungal pathogenesis at temperatures ranging from mammalian conditions to those of natural fungal environments. Of note is that a variety of other traits have been found to play a role in virulence at lower temperatures (Table 1).

## What System Is Better for Studying Phagocytosis?

If we want to reveal the phagocytosis process, unicellular organisms like amoeba or slime molds such as *D. discoideum* are amenable for such studies. For example, amoebae such as *A. castellanii* phagocytose fungi like *C. neoformans*, *Saccharomyces cerevisiae*, and *C. albicans*. These amoebae envelope the fungal cell into a vacuole [1]. Interestingly, during the interaction between *A. castellanii* or insect hemocytes and fungi, fungal structures such as capsule and phospholipase activity provide protection, as they would in mammalian macrophages [1].

Also, model hosts like *G. mellonella* and *D. melanogaster* utilize phagocytic cells as part of their host defense. For example, an indicator of the active response of *G. mellonella* to fungal infections is the number of hemocytes, the phagocytic cells for *G. mellonella*, present after pathogen infection. There is an inverse relationship between the virulence of the invading fungi and the number of hemocytes. Introduction of pathogenic strains does not garner an increase in hemocytes. However, infecting larvae with non-pathogenic strains of fungi causes a release of hemocytes and therefore an increase in the number of hemocytes in the hemolymph [11]. Interestingly, *C. albicans* evade and escape hemocytes utilizing hyphae, similar to the action taken against mammalian phagocytes. On the contrary, phagocytosis is not part of *C. elegans* response to infection. Also, although *D. discoideum* might be too small to phagocytose some of the larger fungi, this host has contributed to understanding phagocytic processes through the study of actin cytoskeleton, an integral part of the phagocytotic process [12].

## Which System Is More Appropriate for Studying Antimicrobial Compounds?

The use of model hosts can facilitate the study of existing and discovery of new compounds. The model systems that have been used most frequently in the field of drug discovery are *C. elegans* and the insects *D. melanogaster* and *G. mellonella*. The nematode *C. elegans* has been the most amenable to the screening process in search of new antifungal compounds due to its small size and use in liquid assay format, making it ideal for implementing high-throughput assays utilizing automation [13]. During the application of this method, liquid infection assays are set up in 96- or 384-well plate formats. Automated systems can supply the plates with the liquid media, nematodes, and specific quantities of compounds [14]. Thus, the testing of thousands of candidate compounds is accelerated. The process can identify not only antifungal compounds, but also those with immunomodulatory effects that bolster the immune response, effectively inhibiting the fungal infection.

Insects can be used for the study of smaller compound libraries. An insect model host system used for the discovery of new antifungal drugs is *G. mellonella*. The substance astemizole, which is an antihistamine drug, found to be active in combination with fluconazole, against *C. neoformans*. Even combination of a few (2–3) compounds can be studied in the survival of infected larvae [15]. In addition, the compound lovastatin was evaluated using *D. melanogaster* as a model for infection from zygomycetes. Lovastatin was active against the fungi *Rhizopus homothallicus*, *Rhizopus oryzae*, *Mucor circinelloides*, and *Cunninghamella bertholletiae* [16]. When *D. melogaster* is utilized as an infection model, a candidate antifungal compound is ingested by the host. However, the exact quantity of the consumed compound is unknown. In the case of *G. mellonella*, a standardized concentration of the compound is delivered via injection. Although more accurate in quantification, the process is time consuming.

## Conclusion

There are several hosts used to model infections ranging from single cell protozoa to insects. For the best interrogation into host–pathogen interactions, researchers can select from a variety of invertebrate model hosts. However, no single model host can answer all scientific questions. The selection of the appropriate host should be based on the virulence trait or the host response under study and the financial, space, and time commitment required (for example, *D. melanogaster* requires incubators and a “fly room”, *C. elegans* requires incubators and microscopes however *G. mellonella* can be used in almost any laboratory). Importantly, scientists can also use the “multi host” approach and implement multiple complementary infection models as they try to understand the various mechanisms in the fungal arsenal to establish an infection, evade or cope with host defenses, and grow and reproduce within the confines of another organism.
